# 3D photon impact determination in monolithic crystals based on autocorrelation filters and RTP methods

**DOI:** 10.1186/2197-7364-2-S1-A5

**Published:** 2015-05-18

**Authors:** Pablo Conde, Antonio Gonzalez, Marco Bettiol, Andrea Fabbri, Roberto Pani, José María Benlloch Baviera, Albert Talens Aguilar, Liczandro Hernandez, Filomeno Sanchez

**Affiliations:** Institute for Instrumentation in Molecular Imaging, I3M-CSIC, Valencia, Spain; Department of Molecular Medicine, Sapienza University of Rome, Italy

In PET detectors based on monolithic scintillators, the photon impact position can be estimated from the light intensity distribution (LD) on the photodetector pixels. Typically, there is a poor estimation of the interaction positions towards the edges when linear algorithms such as Center of Gravity (CoG) are used. We present a novel method to determine the interaction coordinates in thick monolithic crystals filtering the digitized LDs from each gamma-event by means of an autocorrelation filter and the raise to power (RTP) positioning algorithm to reduce the border effects. The experimental setup was based on two detector blocks based on monolithic LYSO scintillator crystals (50x50x20 mm^3^). Each crystal is coupled to a SiPMs array as 12x12 photosensors and an electronic readout that outputs information of each SiPM row and column. Between the detector blocks, a collimated array of 9x9 ^22^Na sources, separated 5 mm each other, was placed. The optimum power to use in the RTP positioning algorithm was determined using the third order intercept point (IP3) from plots of the measured coordinates versus known positions. After applying the autocorrelation and RTP fifth to the data, we found an improvement of the spatial resolution from 2.5 mm when CoG is used, to 1.2 mm in the crystal center region. In this work we show how to accurately resolve 3D photon impact coordinates in thick monolithic crystals using autocorrelation filters merged with RTP methods. After applying the new approach it is possible to accurately resolve impacts close to the entrance of 20 mm thick LYSO scintillators. The reached spatial resolution at any photon depth of interaction is comparable with state-of-the-art crystal array approaches with the advantage of the proposed work to also provide continuous depth of interaction information.

